# The Hip Functional Retrieval after Elective Surgery May Be Enhanced by Supplemented Essential Amino Acids

**DOI:** 10.1155/2016/9318329

**Published:** 2016-03-24

**Authors:** Eleonora Baldissarro, Roberto Aquilani, Federica Boschi, Paola Baiardi, Paolo Iadarola, Marco Fumagalli, Evasio Pasini, Manuela Verri, Maurizia Dossena, Arianna Gambino, Sharon Cammisuli, Simona Viglio

**Affiliations:** ^1^Dipartimento di Medicina Fisica e Riabilitativa, Salvatore Maugeri Foundation, Centro Medico di Nervi, IRCCS, Fondazione Maugeri, Via Missolungi 14, 16167 Nervi, Italy; ^2^Dipartimento di Biologia e Biotecnologie, Unità di Biochimica, Università di Pavia, Via Ferrata 2, 27100 Pavia, Italy; ^3^Dipartimento di Scienze del Farmaco, Università di Pavia, Via Taramelli 12, 27100 Pavia, Italy; ^4^Direzione Scientifica Centrale, IRCCS, Fondazione Maugeri, Via Boezio 28, 27100 Pavia, Italy; ^5^Istituto Scientifico di Lumezzane, IRCCS, Fondazione Maugeri, Via Mazzini 129, 25065 Lumezzane, Italy; ^6^Consorzio per Valutazioni Biologiche e Farmacologiche, Via Porta 12, 27100 Pavia, Italy; ^7^Dipartimento di Medicina Molecolare, Unità di Biochimica, Università di Pavia, Via Taramelli 3/B, 27100 Pavia, Italy

## Abstract

It is not known whether postsurgery systemic inflammation and plasma amino acid abnormalities are still present during rehabilitation of individuals after elective hip arthroplasty (EHA). Sixty subjects (36 females; age 66.58 ± 8.37 years) were randomized to receive 14-day oral EAAs (8 g/day) or a placebo (maltodextrin). At admission to and discharge from the rehabilitation center, serum C-reactive protein (CRP) and venous plasma amino acid concentrations were determined. Post-EHA hip function was evaluated by Harris hip score (HHS) test. Ten matched healthy subjects served as controls. At baseline, all patients had high CRP levels, considerable reduction in several amino acids, and severely reduced hip function (HHS 40.78 ± 2.70 scores). After treatment, inflammation decreased both in the EAA group and in the placebo group. Only EAA patients significantly improved their levels of glycine, alanine, tyrosine, and total amino acids. In addition, they enhanced the rate of hip function recovery (HHS) (from baseline 41.8 ± 1.15 to 76.37 ± 6.6* versus* baseline 39.78 ± 4.89 to 70.0 ± 7.1 in placebo one; *p* = 0.006). The study documents the persistence of inflammation and plasma amino acid abnormalities in post-EHA rehabilitation phase. EAAs enhance hip function retrieval and improve plasma amino acid abnormalities.

## 1. Introduction

Elective hip arthroplasty (EHA) is a surgical technique used for patients with damaged hip joints after degenerative osteoarthritis or an injury. One and half million EHA operations are carried out all over the world each year [1]. In Italy, there are about 100,000 annual EHA operations at a cost of approximately 1.3 billion euros, to which 500 million euros should be added for subsequent rehabilitation (rehab) treatment. The total economic burden of EHA + rehab is 1.6% of the national annual budget of the health care system [1].

EHA and post-EHA rehab allow patients to regain physical ability and improve their quality of life. However, a number of factors may hinder both the duration and outcome of rehabilitation, including preoperative functional state, postoperative anemia, and any infection or metabolic alterations. Among the latter, the possible persistence of inflammation and alterations of plasma amino acids [2–4] can lengthen functional restoration time, reduce clinical outcome, and increase the need for additional rehab [5]. Inflammation can increase the risk of local (wounds) and systemic complications, whereas low plasma amino acids can hinder tissue repairing, given their essential anabolic role for wound healing [6] and the recovery of limb skeletal mass. Limiting these postsurgery alterations could accelerate and enhance the retrieval of operated hip-joint dysfunction.

Here, we have formulated two hypotheses. Firstly, rehab subjects after EHA could have alterations of circulating amino acids from persistent inflammation. To test this hypothesis, we measured plasma amino acid levels and inflammation markers. Secondly, this study investigated whether the administration of essential amino acids (EAAs) could enhance the recovery of hip-joint dysfunction of the operated hip and correct/limit plasma amino acid alterations. The rationale for this choice was based on the fact that these substrates are well-known to enhance body and muscle protein synthesis (hence tissue accrual) [7], even during acute inflammation [8].

The ultimate purpose of this study was to stimulate further research on the topic and to provide added recommendations for rehabilitation management.

## 2. Methods

### 2.1. Study Design and Participants

Sixty-eight subjects at 17 ± 1.12 days after elective hip arthroplasty (EHA) were recruited at admission to the rehabilitation center. After operation and before the admission to our rehabilitation department, patients stayed an average of 12 days in orthopedic department to achieve clinical stabilization and to start training for erected posture and gait. The patients were selected on the basis of the type of surgical access they had had (lateral approach) in order to establish a more uniform recovery time. The causes for EHA included aseptic necrosis of the femoral head (5%), rheumatoid arthritis (1.6%), congenital hip dysplasia (1.6%), and grade 3 and 4 osteoarthritis (91.8%) diagnosed through preoperatory X-ray. Eight subjects were excluded due to diabetes on insulin and/or oral hypoglycemic drugs (*n* = 5), hyperthyroidism (*n* = 1), chronic renal disease (*n* = 2), and all conditions which could alter plasma amino acid levels. As a result, a total of 60 patients were studied.

On the second day after admission, patients underwent the following procedures.

*(a) Anthropometric Measurements.* They are body weight (BW; kg) and height (m). Body mass index (BMI) was calculated as kg/m^2^. 

*(b) Biohumoral Variables*

(1) Routine variables, including serum total protein (g/dL) and protein electrophoresis.
(2) C-reactive protein (CRP) as a marker of systemic inflammation (using an immune-turbidimetric method. Normal value is <0.8 mg/dL).
(3) Plasma amino acids: the concentration of free amino acids in the plasma was measured using an AminoQuant II amino acid analyzer, based on the HP 1090 HPLC system, with fully automated precolumn derivatization. Both orthopthalaldehyde (OPA) and 9-fluorenyl-methyl-chloroformate (FMOC) reaction chemistries were used, according to the manufacturer's protocol. Measurements were made by injecting 1 *μ*L of the derivatized mixture and measuring absorbance simultaneously at 338 and 262 nm. Plasma concentrations were expressed as *μ*mol/L. Baseline plasma amino acids were compared to those of 10 healthy subjects matched for sex distribution (4 males), age (69 ± 7.5 years), and body mass index (27.5 ± 3.5 kg/m^2^).

*(c) Clinical-Functional Status*. This was measured using the Harris hip score (HHS) [9]. This is a test consisting of several domains relative to pain, limp, support, distance walked, sitting, entering public transportation, stairs, putting shoes and socks on, absence of deformity, and a range of motion. The test gave outcome classifications as poor (<70 scores), sufficient (70–79 scores), good (80–89 scores), or excellent (90–100 scores) [10].

*(d) Nutritional Intake*. A 3-day alimentary diet was kept by the rehab nurses, trained* ad hoc*. The nurses recorded the type and weight of cooked or uncooked food selected by patients from the hospital catering menu on a diet sheet for 3 days before and after the patients' meals [11].

Nutritional analysis on the amount of food actually ingested was used to calculate actual ingested calories and macronutrients [12]. Nutritional intake was analyzed only at baseline.

Patients were then assigned to the treatment or placebo according to a randomized allocation procedure established before patient admission to our rehabilitation department. A randomization list was generated using SAS statistical software, A and B being the identifiers of the blinded treatment. The list was made available to both the physiatrist (E. B.) and the hospital pharmacist. The physiatrist allocated patients sequentially to treatment A or B according to the randomization list. The author of the article (A. R.) who interpreted all the results and the physiatrist (S. C) who evaluated the clinical-functional state (HHS) were blinded to the patient allocation. The treatment consisted of 14-d EAA supplementation. Eight g/d (2 packages) of EAAs (Aminotrophic, Professional Dietetics, Milan, Italy) or a placebo (maltodextrin) was supplied. One package (4 g) of EAAs contained the essential leucine (1.250 g), lysine (0.65 g), isoleucine (0.625 g), valine (0.625 g), threonine (0.35 g), phenylalanine (0.1 g), methionine (0.05 g), and tryptophan (0.02 g) as well as cysteine (0.15 g), tyrosine (0.03 g), and histidine (0.15 g). The 14-day treatment was dictated by the rehabilitation center policy, which permitted patients to stay for a maximum of 20 days.

With regard to the placebo, we decided not to use isonitrogenous nonessential amino acids as, under physiological conditions, these amino acids are inferior to essential amino acids in boosting protein synthesis or limiting protein breakdown. The use of maltodextrin as a placebo would have been a more appropriate choice since this carbohydrate, like essential amino acids [11, 13, 14], potentially exerts an immunological effect [9]. An imbalanced immunological effect between placebo and EAAs would have potentially caused imbalanced infection rates with consequent intergroup differences in altered systemic and muscle protein turnover. Thus, according to this reasoning, maltodextrin (placebo) and EAA groups would have been associated with similar postsurgery infection rates affecting plasma amino acid concentrations and functional recovery.

*(e) The Rehabilitation Protocol.* This protocol aimed to restore complete functional recovery of the altered body statics and the resumption of a normal walking pattern. The rehabilitation program included 2 daily therapy sessions lasting 45 minutes each (morning and afternoon), with patients assisted by the same physiotherapist. The entire rehabilitation cycle encompassed 24 sessions. The rehabilitation program consisted of the following:

1. Passive mobilization of hip movements in triple flexion (hip, knee, and ankle).
2. Stretching the adductor and flexor muscles of the operated limb.
3. Hip extension using isotonic contraction of the gluteus.
4. Isotonic contraction of the quadriceps muscles against a resistance of 1 kg.
5. Assisted gait training with the use of walking sticks and training for the stairs.
6. Maintenance of cardiorespiratory capacity.

Within 2 days before patient discharge, all procedures except for nutritional intake were repeated. The local ethics scientific committee approved the protocol (# 118 in the Maugeri Foundation Scientific Annals 2011, page 432) after the subjects gave their informed consent.

### 2.2. Statistical Analysis

Descriptive statistics were carried out for all recorded variables, reporting mean and standard deviations for quantitative variables and distribution frequencies for qualitative variables. Chi-squared test was used for categorical variables. Wilcoxon signed rank test was used to compare the difference in HHS of the entire patient population between admission to and discharge from rehabilitation institute. Paired Student's* t*-test was used to compare differences between admission and discharge in patient's plasma amino acids. Baseline variable differences between the randomized patient subgroups were tested, when appropriate, by unpaired Student's* t*-test, chi-square, and Mann-Whitney test.

Repeated measures analysis of variance tested the over time changes in plasma amino acids of the randomized subgroups. Kruskal-Wallis test was used to compare the over time gains of HHS in randomized EAA and placebo subgroups. Statistical significance was set at *p* < 0.05.

## 3. Results

### 3.1. Baseline Characteristics of Patient Population at Admission to Rehab

Table 1 shows the demographic, anthropometric variables, nutritional intake, biohumoral variables, and clinical-functional tests of the entire patient population at admission and at discharge. Generally, patients were slightly overweight, with none of them being underweight. Their nutritional intake was normal according to the recommended allowance of our national institute of nutrition. Thirty-five percent of patients (*n* = 21; 12 in EAA group) were on antibiotic therapy for infection (mainly of urinary tract). The used antibiotics were amoxicillin (4 in EAA group, 6 in placebo group) or levofloxacin (6 in placebo group, 5 in EAA group). Seventy percent (*n* = 42; 23 in EAA group) had received blood transfusions (1 blood unit; only 1 patient, in the placebo group, had received 2 blood units) during their acute stage.

All patients had systemic inflammation as indicated by elevated serum CRP, which was more than 25 times higher than the normal maximal value. The erythrosedimentation rate (ESR) and the positive reactants of inflammatory response (serum alpha 1 and alpha 2 globulin concentrations) were above normal, whereas negative proteins (albumin and transferrin) were normal. All patients had mild anemia (Table 1).

Functionally, the patients had severe reduction of hip function as indicated by their HHS test (40.78 ± 2.70 scores). Within the test, the domain relative to pain showed that patients suffered with moderate pain (19.5 ± 1.7 scores).

Plasma amino acid levels can be seen in Table 2. Compared to healthy subjects, patients had significantly lower plasma levels of asparagine (−14.4%), serine (−41%), glycine (−37%), glutamine (−79.5%), tyrosine (−28%), essential phenylalanine (−27%), and lysine (−41%). On the contrary, the levels of arginine (+122%), threonine (+67.7%), and citrulline (+130.5%) were higher in patients than in controls. Table 2 shows that, at discharge from rehab, patients maintained abnormal plasma levels of amino acids compared to healthy controls. Nevertheless, at discharge, plasma levels of glutamine (*p* < 0.05) and citrulline (*p* < 0.05) were significantly improved in patients.

### 3.2. After Randomization

*(a) Differences in Baseline Characteristics between EAA and Placebo Groups.* After randomization (Table 3), the EAA and placebo groups were similar in nutritional status, dietary intake, and distribution in infection complications (40% in placebo, 30% in EAAs; n.s.).

The EAA group was different from the placebo one in the time from index event (*p* < 0.05). Compared to the placebo group, the EAA subjects had lower serum transferrin (*p* = 0.04) and circulating total proteins (*p* = 0.04). Other biohumoral variables including CRP and plasma amino acids were similar. Hip dysfunction was similar between the two patient groups (39.78 ± 4.89 scores in placebo* versus *41.80 ± 1.15 scores in EAA group; n.s.). Within the test, all domains including that which is relative to pain were similar between placebo and EAA subjects.

*(b) After 14-d Treatment*. All patients randomized to receive EAAs or placebo completed the study.

During rehab, the inflammation rate decreased similarly for both groups (CRP −7.8 ± 4 mg/dL in placebo* versus* −9.2 ± 6.5 mg/dL in EAA group, interaction *p* = 0.6; ESR −11.03 ± 5.4 mm in placebo* versus* −13.02 ± 6.4 mm in EAA group, interaction *p* = 0.7). Blood Hb and serum transferrin increased by +0.84 ± 0.3 g/dL in placebo* versus* +0.87 ± 0.19 g/dL in EAA group, interaction *p* = 0.8 for Hb; +1.34 ± 2.5 mg/dL in placebo* versus* +11.45 ± 3.1 mg/dL in EAA group, interaction *p* = 0.005 for transferrin. All other biohumoral variables did not show significant changes in both intra- and intergroups.

As can be seen from Table 4, compared to controls, EAA patients improved over time the levels of glycine (interaction *p* = 0.001), alanine (interaction *p* = 0.02), tyrosine (interaction *p* = 0.05), total AAs (interaction *p* = 0.02), and total EAAs (interaction *p* = 0.05). Arginine in the EAA group decreased towards the control value. The over time changes of other amino acids were similar between the two groups.

**Figure 1:**
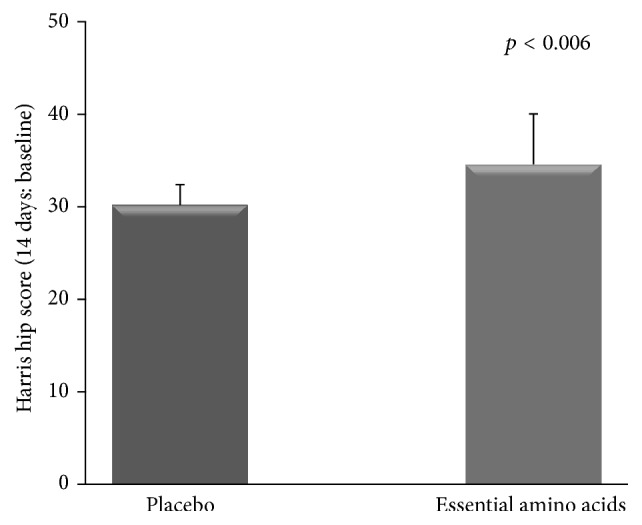
Time courses of improvements of clinical-functional test in the two postsurgery subgroups. The plot shows the superiority of essential amino acids in enhancing hip function recovery.

All patients improved hip dysfunction (from baseline 48.78 ± 2.70 to 73.18 ± 7.1 scores; *p* < 0.001) but the EAA patients improved more (from baseline 41.8 ± 1.15 to 76.37 ± 6.6 scores* versus *39.78 ± 4.89 to 70.0 ± 7.10 scores) (*p* < 0.006); (Figure 1). In the HHS test, the EAA group reduced pain considerably more (from 20 ± 0 to 39.2 ± 5.59 scores) than the placebo patients did (from 18.7 ± 3.4 to 32.4 ± 6.2 scores) (*p* = 0.01). The over time changes in the other HHS items resulted similar between placebo and EAA groups (Table 5).

## 4. Discussion

This study shows that, in the rehabilitation phase of EHA, patients may have abnormal plasma levels of several amino acids that could be associated with systemic inflammation and may persist until the end of rehabilitation. While supplementation with EAAs may optimize post-EHA rehabilitation by enhancing the functional retrieval of hip-joint function, EAAs cannot correct amino acid abnormalities.

### 4.1. Baseline Characteristics of the Patients at Admission to Rehab

Inflammation is clearly a sequela of surgical trauma [15], although the inflammation rate is less than after surgery for traumatic fracture or in critical care patients [16]. A number of factors including pre- and postsurgery functional status, analgesia [17], surgical trauma with consequent neuroendocrine-mediated hypercatabolic process, and inflammatory responses could all explain the profound changes in plasma concentration of several amino acids. These include glycine and lysine which, together with proline (not determined in this study), are the most abundant amino acids of collagen, the principal protein of wound repair [18].

The reduced availability of these amino acids may, therefore, increase the risk of impaired and/or delayed wound repair and low quality of scar formation. The low glutamine level in patients combines with glycine and lysine in negatively influencing wound repair. Indeed, glutamine is an important fuel for fibroblast activities [19]. Reduced plasma glutamine concentrations in this study are in keeping with the finding that surgery reduces this amino acid, with such reduction lasting at least 30 days after the operation [20]. Low glutamine reduces the efficiency of immune response [21], for instance, by preventing expression of membrane receptors and cytokines, thus perpetuating systemic inflammation. Since arginine levels usually decreased after trauma, the finding of its increase in plasma was unexpected. We have speculated that this increase could be the result of glutamine metabolism at the intestinal-renal axis [22]. Increased arginine availability can potentially aid wound repair by (i) increasing collagen amount and growth of hormone synthesis [23]; (ii) improving pancreas insulin release and peripheral tissue insulin sensitivity [24]; and (iii) regulating lymphocyte function [25] and memory [26], especially of T-cells, thus reducing infectious complications. The low serine levels observed in our patients reflect increased consumption following increased protein turnover after operation. Indeed, serine participates in the biosynthesis of pyrimidines and purines and is a precursor of glycine and cysteine, both of which are important immunogenic amino acids.

The coexistence of amino acid abnormalities with the patients' normal protein intake indicates that the intake of protein (1 g/kg body weight) is not enough to correct alterations of amino acids. This raises the question of whether, in post-EHA, the observed altered amino acids could behave as semiessential amino acids.

In synthesis, rehabilitative patients after EHA may have an abnormal plasma amino acid profile, potentially increasing the risk for poor quality of wound repair.

### 4.2. Effects of Nutritional Intervention

After randomization, the two patient groups had different amounts of circulating transferrin and total proteins. Given the similarities for the other measured variables, these differences could be due to both different preoperative protein status and different postoperative metabolic response rates. This is suggested by both the time from index event and the trend to stay longer in acute setting observed in EAA patients.

The study shows that a supplementation with EAAs improved the levels of the circulating amino acids and enhanced hip function retrieval. The treatment with EAAs probably gave the patients a more balanced protein turnover. This is suggested by both the maintenance of branched chain amino acid (BCAA, leucine, isoleucine, and valine) concentrations and the improved concentration of glycine, threonine, and alanine, with normalization of arginine. This was not the case for controls.

However, the EAA treatment failed to normalize plasma glutamine. This would suggest that body requirements for this amino acid were higher than that provided both exogenously by diet and endogenously by essential BCAA metabolism [27].

Several mechanisms may explain the enhanced retrieval rate of hip-joint dysfunction associated with EAA supplementation. One mechanism is related to the effects of EAAs on wound repair. The phases of wound healing (i.e., inflammation, proliferation, and remodeling) require high EAA and semiessential glutamine and arginine [6] availability because they are metabolically characterized by* de novo* synthesis of an enormous amount of peptides and proteins. To support this, a very recent study has reported that the infusion of amino acid mixtures (including the essential ones) has anabolic effect during acute inflammation [8].

Another mechanism underlying the beneficial effects of EAA on hip retrieval may be the potentiation of the increase of muscle mass and strength of the operated limb, damaged by factors including surgical stress, reduced or absent muscle contraction, being bed-ridden, unloading, and inflammation. In support of this, the 12-week administration of 7.5 g EAAs (very similar to the amount used here) to older women was shown to increase muscle mass (as measured by Dexa), while they failed to improve clinical outcomes such as knee extension and handgrip [28]. This is in contrast with the improved hip dysfunction found here. These conflicting data may be explained by the different muscle status that was probably damaged in our patients, whereas it was not presumably damaged in the previous study. This is reinforced by two factors. First, EAAs induce muscle mass accrual and function even during bed-rest in the elderly [29]. EAAs promote muscle protein synthesis and accrual by stimulating the mammalian target of the rapamycin (mTOR) pathway, which is an essential step for the regeneration of damaged muscles [30]. Supplemented EAAs may be synergistic with rehabilitative exercises in increasing the mass and function of damaged muscles [31], as each of them individually stimulates the hormone insulin growth factor-1 (IGF-1) that increases muscle protein synthesis and decreases protein degradation [32]. In addition, EAAs significantly reduce the exercise-induced impairment of muscle protein synthesis [30].

A further mechanism primed by EAAs to improve hip retrieval may be the attenuation of muscle weakness and soreness [30], which are symptoms of muscle damage. Overall, post-EHA patients, including those in the current study, suffer from muscle soreness, pain, and weakness, particularly after each session of physical therapy. A possible reduction in muscle soreness could in part explain the reduced domain relative to pain observed here, which was more pronounced in the EAA group. Theoretically, we cannot totally exclude some influence of supplemented EAAs on the central nervous system, contributing, in turn, to reduction of the pain domain within the HHS test. This is because amino acids are largely consumed by nervous system cells and several of these are precursors of neurotransmitters.

A preferential/prevalent use of supplemented EAAs in wound areas and regional muscles could also help explain the coexistence of plasma amino acid alterations and EAA's failure to correct these alterations as well as better retrieval of hip-joint dysfunction.

## 5. Study Limitations

The study has several limitations that need to be addressed in future investigations.

These results need to be confirmed in a larger sample size, which also takes into account the changes of these variables in relation to gender and patient body composition. This requires a well-planned powered study. Understanding the patients' preoperative physical dysfunction rate would have strengthened the discussion. Pre- and postoperative assessment of muscle mass and strength could help us predict the gain in physical recovery better.

## 6. EAAs and Post-EHA Rehabilitation: Clinical Considerations

Notwithstanding these limitations, we believe this study can provide some useful suggestions for clinical practice.

The persistence of unmet metabolic alterations during the rehabilitation phase of EHA may predispose patients to a persistence of impairment and functional limitations at short- and long-term follow-up [33]. Thus, EAA supplementation in the early postoperative phase (operation interval < 8 weeks) [34] may help patients to reduce the rate of persisting hip dysfunction. EAA-induced pain reduction allows patients not to discontinue the daily physical therapy session. It is interesting to note that pain was not improved in patients on intensified home-based exercise after total hip replacement [35] or in subjects on aquatic physiotherapy [36], even though this type of physiotherapy has given conflicting results in terms of pain relief [37]. In the light of the fact that evidence to build up a detailed evidence-based exercise protocol [34] in the rehabilitation phase of post-EHA is insufficient, we think that the addition of EAAs to post-EHA early rehabilitation might be particularly advantageous for both physiotherapist and patient. Rehab patients after EHA could be supplemented with EAAs and glutamine for at least three reasons. First, quick reacquisition of physical autonomy by elderly subjects is very important to reduce the risk of frailty syndrome. Second, the high prevalence of postoperative infections (35% of the study population) increases the need for EAA availability. The reason for adding glutamine to the EAA mixture is that the lack of the amino acid may counteract the anabolic effect promoted by EAAs by inducing muscle proteolysis [38]. Third, given that the entire population of patients was discharged with anemia, continuing EAA supplementation after rehab may increase the synthesis of hemoglobin.

In conclusion, it could be useful to provide patients with a supplementation of EAAs, both during postexercise recovery to stimulate muscle protein synthesis [30] and during the day to reduce muscle soreness, the intensity and duration of which could compromise efficient rehabilitation.

## 7. Future Studies

This investigation would prompt us to look at two different research areas concerning changes in the dose and/or composition of the formula we used. For example, EAAs need to be tested at a dose higher than 8 g/d and/or for longer, to document whether plasma amino acid alterations may be corrected and functional recovery further enhanced. In addition, it could be clinically important to seek whether increasing the formula used here, that is, the amount of lysine and phenylalanine and adding the nonessential glycine, serine, asparagine, and glutamine, could further improve retrieval of postsurgery hip function. This could be particularly important for those patients who return home for full-hip function recovery and for subjects who, for any reason, do not have access to a post-EHA rehabilitation program.

## 8. Conclusions

This study shows that recovery of hip dysfunction after elective surgery of EHA may be enhanced by short-term supplemented essential amino acids which, however, fail to correct plasma amino acid alterations.

## Figures and Tables

**Table 1:** Demographic, anthropometric variables, nutritional intakes, biohumoral variables, and clinical-functional test of the entire patient population at admission to our rehab institute.

	Admission	Discharge	*p*
Age (yrs)	66.58 ± 8.37	—	n.s.
M/F	24/36	—	n.s.
Body weight (kg)	74.5 ± 14.5	73.9 ± 14.5	n.s.
Body mass index (BMI) kg/m^2^	26.6 ± 4.1	26.5 ± 4.05	n.s.
Time from index event (days)	17.05 ± 1.12	34 ± 2.0	n.s.
Length of stay (days)	16.9 ± 1.0	—	n.s.
*Daily nutritional intakes*			
Energy (kcal)	2123 ± 325	—	n.s.
kcal/kg	28.5 ± 2.7	—	n.s.
Carbohydrates (g/kg)	3.9 ± 0.6	—	n.s.
Proteins (g/kg)	1.01 ± 0.3	—	n.s.
Lipids (g/kg)	1.01 ± 0.35	—	n.s.
*Blood biohumoral variables*			
Glucose (mg/dL; n.v.^*∗*^ 70–110)	84.6 ± 14.8	83.4 ± 12	n.s.
Urea (mg/dL; n.v.^*∗*^ 16–38)	35.1 ± 8.8	34.6 ± 7.7	n.s.
Creatinine (mg/dL; n.v.^*∗*^ 0.6–1.1)	0.74 ± 0.09	0.76 ± 0.12	n.s.
Erythrocyte sedimentation rate (mm/1 hr; n.v.^*∗*^: <12)	47.9 ± 25.9	40.4 ± 24.9	n.s.
C-reactive protein (mg/dL; n.v.^*∗*^ <0.8)	20.4 ± 19.6	8.21 ± 11.1	n.s.
Hemoglobin (g/dL; n.v.^*∗*^ >13 males and >12 females)	10.4 ± 1.09	11.3 ± 1.09	<0.02
Iron (*μ*g/dL; n.v.^*∗*^ 40–130)	40.4 ± 19	48.5 ± 17.7	n.s.
Ferritin (ng/mL; n.v.^*∗*^ 30–230 males and 10–120 females)	257.4 ± 201.8	235 ± 182	n.s.
Transferrin (mg/dL; n.v.^*∗*^ 180–240)	203 ± 32	209 ± 30.6	n.s.
Albumin (g/dL; n.v.^*∗*^ 3.5–5.5)	3.55 ± 0.37	3.79 ± 0.33	n.s.
Total protein (g/dL; n.v.^*∗*^ 6–8)	6.24 ± 0.50	6.49 ± 0.47	n.s.
*α*1-globulin (% total protein; n.v.^*∗*^ 2.9–4.9)	6.96 ± 1.28	5.49 ± 0.71	n.s.
*α*2-globulin (% total protein; n.v.^*∗*^ 7.1–11.8)	12.45 ± 2.2	11.33 ± 2.06	n.s.
*Clinical-functional test* ^#^			
Harris hip score	40.78 ± 2.70	73.15 ± 7.52	<0.0001

Data are expressed as mean ± standard deviation. ^*∗*^Normal value.

Statistical analysis: paired *t*-test; n.s. = not significant.

^#^Wilcoxon signed rank test.

**Table 2:** Venous plasma concentration (*μ*mol/L) of amino acids measured in the entire patient population both at admission to and discharge from our rehab institute. For comparison, plasma amino acid levels in healthy subjects were reported.

	Healthy subjects (*n* = 10)	Patients (*n* = 60)	
		Admission	Discharge (*p*)
Aspartate	19 ± 9	21.9 ± 11.6	16 ± 9
Glutamate	95 ± 63	154 ± 93	128.6 ± 32
Asparagine	23 ± 9	19.7 ± 9.2^*∗*^	19.1 ± 11.2^■^
Serine	111 ± 31	65.8 ± 33^○^	50.8 ± 15.2^○^
Glutamine	483 ± 100	99.4 ± 32^○^	120.5 ± 28^#○^
Histidine	62 ± 12	68.6 ± 35.6	66.5 ± 24
Glycine	227 ± 80	143 ± 107^*∗*^	122 ± 23.5^○^
Threonine	118 ± 32	198 ± 104	173.7 ± 62.9
Citrulline	8.5 ± 2.1	19.6 ± 9.4	22.3 ± 9.7^#^
3-Methylhistidine	3.24 ± 0.87	4.86 ± 2.5	6.4 ± 4.1
Alanine	385 ± 92	307 ± 82	298 ± 89^■^
Arginine	62 ± 20	138 ± 60^■^	160 ± 81.40^■^
Tyrosine	65 ± 14	47 ± 16^■^	48.7 ± 16.7^*∗*^
Cysteine	—	48 ± 28.5	50 ± 16.8
Valine	249 ± 58	202 ± 74	203 ± 69
Methionine	21.5 ± 5.2	24.8 ± 15.7	18.1 ± 10
Tryptophan	70 ± 33	70.3 ± 33	59 ± 28
Phenylalanine	70 ± 30	51.20 ± 20	50.5 ± 20^*∗*^
Isoleucine	68 ± 16	63.6 ± 26	61 ± 21
Leucine	128 ± 28	105 ± 49	102 ± 35.8
Lysine	180 ± 36	107 ± 57^*∗*^	96 ± 21^○^
Total AAs	2493.5 ± 652.4	1960 ± 890.5^*∗*^	1872 ± 628.3^■^
Total EAAs	904.5 ± 238	822 ± 379	821.5 ± 268

Data are expressed as mean ± standard deviation.

Statistical analysis:

(a) unpaired *t*-test between patients and healthy subjects at both admission to and discharge from rehab institute; ^○^ *p* < 0.001; ^■^ *p* < 0.02; ^*∗*^ *p* < 0.05;

(b) paired *t*-test between patient discharge and admission: ^#^ *p* < 0.05.

**Table 3:** Demographic, anthropometric variables, nutritional intakes, biohumoral variables, and clinical-functional test of the admitted patients after randomization.

	Placebo	EAAs	*p*
Age (yrs)^†^	65.6 ± 9.50	67.9 ± 7.3	0.2
M/F^‡^	10/20	14/16	0.3
Body weight (kg)^†^	73.0 ± 12.2	75.9 ± 15.9	0.3
Body mass index (BMI) kg/m^2^^†^	27.5 ± 3.9	29.8 ± 4.2	0.07
Time from index event (days)^†^	16.0 ± 1.1	17.9 ± 1.2	<0.05
Length of stay (days)^†^	15.2 ± 1.2	17 ± 0.8	<0.08
*Daily nutritional intakes* ^†^			
Energy (kcal)	1980 ± 285	2210 ± 441	0.3
kcal/kg	27.1 ± 2.1	29.1 ± 4.33	0.5
Carbohydrates (g/kg)	3.7 ± 0.4	4.1 ± 0.8	0.8
Proteins (g/kg)	1.1 ± 0.4	0.9 ± 0.2	0.6
Lipids (g/kg)	0.95 ± 0.25	1.15 ± 0.40	0.8
*Blood biohumoral variables* ^†^			
Glucose (mg/dL; n.v.^*∗*^ 70–110)	82.2 ± 16.5	87.0 ± 15.5	0.6
Urea (mg/dL; n.v.^*∗*^ 16–38)	34.7 ± 10.9	35.7 ± 8.1	0.2
Creatinine (mg/dL; n.v.^*∗*^ 0.6–1.1)	0.73 ± 0.08	0.74 ± 0.09	0.2
Erythrocyte sedimentation rate (mm/1 hr; n.v.^*∗*^: <12)	45.3 ± 24.2	49.4 ± 28	0.4
C-reactive protein (mg/dL; n.v.^*∗*^ <0.8)	16.4 ± 11.7	20.5 ± 18.1	0.5
Hemoglobin (g/dL; n.v.^*∗*^ >13 males and >12 females)	10.4 ± 1.2	10.5 ± 0.96	0.1
Ferritin (ng/mL; n.v.^*∗*^ 30–230 males and 10–120 females)	206.7 ± 166	288 ± 212	0.3
Transferrin (mg/dL; n.v.^*∗*^ 180–240)	215 ± 34	191 ± 29	<0.04
Albumin (g/dL; n.v.^*∗*^ 3.5–5.5)	3.62 ± 0	3.57 ± 0	<0.07
Total protein (g/dL; n.v.^*∗*^ 6–8)	6.37 ± 0.51	6.1 ± 0.38	<0.04
*α*1-globulin (% total protein; n.v.^*∗*^ 2.9–4.9)	6.7 ± 1.1	6.97 ± 1.38	0.1
*α*2-globulin (% total protein; n.v.^*∗*^ 7.1–11.8)	11.9 ± 2.5	11.1 ± 2.4	0.1
*Clinical-functional test* ^#^			
Harris hip score	39.78 ± 4.89	41.8 ± 1.15	0.9

Data are expressed as mean ± standard deviation. ^*∗*^Normal value.

Statistical analysis:

(a) ^†^unpaired *t*-test;

(b) ^‡^Chi-square test;

(c) ^#^Mann-Whitney test.

**Table 4:** Plasma amino acid levels (*μ*mol/L) in stroke placebo and treated subgroups at both admission and discharge.

Amino acid	Placebo (*n* = 30)		EAAs (*n* = 30)		Trend over time (interaction; *p* level)
	Admission	Discharge	Admission	Discharge (*p*)	
Aspartate	20.5 ± 8.2	14.3 ± 5.9	24.5 ± 16.7	11.1 ± 7.8	=0.6
Glutamate	177 ± 100	129.6 ± 19.6	143 ± 116	127.6 ± 45	=0.8
Asparagine	21.5 ± 10.6	20.5 ± 11.9	16.7 ± 6.6	17.7 ± 10.5	=0.5
Serine	72 ± 43	51.4 ± 20	64.5 ± 24	50.2 ± 10.3	=0.9
Glutamine	97 ± 33	124.3 ± 21	104.2 ± 31	128.4 ± 33	=0.7
Histidine	74.2 ± 33	72.6 ± 17	58.2 ± 40.1	64.0 ± 31.3	=0.8
Glycine	157 ± 125	121 ± 24.4	118 ± 60	130 ± 22.5	=0.001
Threonine	223 ± 96	177 ± 61.7	151.3 ± 110	170.3 ± 64	=0.1
Citrulline	20.5 ± 9.9	23.1 ± 10	19 ± 9.5	21.5 ± 9.4	=0.6
3-Methylhistidine	5.5 ± 2.9	8.3 ± 6.2	3.78 ± 1.22	4.5 ± 1.9	=0.7
Alanine	356 ± 195	312 ± 65	216 ± 119	284 ± 112	=0.02
Arginine	136 ± 67.7	200 ± 117	144.2 ± 51	119 ± 45	=0.03
Tyrosine	55.3 ± 27	49.3 ± 16.4	43.7 ± 14.9	48.0 ± 17	=0.05
Cysteine	55.1 ± 29.6	55.2 ± 19	34.4 ± 22.3	45.1 ± 14.5	=0.05
Valine	219 ± 71	214.5 ± 59	180.4 ± 77	193.1 ± 80	=0.8
Methionine	28.6 ± 16	19.7 ± 10.4	17.6 ± 14.4	16.5 ± 9.5	=0.6
Tryptophan	76.4 ± 28	62 ± 14.6	59 ± 40.4	56 ± 41.5	=0.8
Phenylalanine	54.4 ± 21	53.3 ± 16.6	45 ± 17	47.6 ± 24	=0.9
Isoleucine	66.5 ± 23	66.3 ± 20	62.1 ± 33	55.2 ± 21.8	=0.8
Leucine	113 ± 46	108 ± 36	98.1 ± 56	95.2 ± 36	=0.9
Lysine	109 ± 52	97 ± 26.6	95.1 ± 51	94.4 ± 15.5	=0.8
Total AAs	2137 ± 1004	1979 ± 603	1699 ± 913	1833 ± 658.5	=0.02
Total EAAs	890 ± 353	798 ± 247	709 ± 400	728 ± 293	=0.05

EAAs = essential amino acids.

Data are reported as mean ± standard deviation.

Statistical analysis: repeated measures analysis of variance.

Trend over time: interaction differences in trends between groups.

**Table 5:** Changes over time of clinical-functional test Harris hip score (HHS) in both placebo and EAA groups.

	Placebo		EAAs		*p* level
	Admission	Discharge	Admission	Discharge (*p*)	
Harris hip score	39.78 ± 4.89	70 ± 7.1	41.8 ± 1.15	76.37 ± 6.6	0.006
*HHS items*					
Pain	18.7 ± 3.4	32.4 ± 6.2	20 ± 0	39.2 ± 5.59	0.01
Support	1.73 ± 0.69	2.7 ± 0.45	1.93 ± 0.37	2.73 ± 0.45	0.7
Limp	4.83 ± 0.91	8.5 ± 1.38	5 ± 0	8.72 ± 1.53	0.5
Distance	1.93 ± 0.36	8 ± 0	2.1 ± 0.55	8 ± 0	0.8
Sitting	3.13 ± 0.5	4.33 ± 0.96	3 ± 0	4.03 ± 1.01	0.7
Public transportation	0	0.56 ± 0.5	0	0.58 ± 0.509	0.9
Stairs	0	2 ± 0	0	2 ± 0	—
Shoe/socks	1.86 ± 0.50	3.06 ± 1.01	2 ± 0	2.69 ± 0.97	0.3
Deformity	4 ± 0	4 ± 0	4 ± 0	4 ± 0	—
ROM	3.51 ± 0.35	4.43 ± 0.33	3.58 ± 0.387	4.44 ± 0.25	0.9

Statistical analysis: Kruskal-Wallis test.

Level of significance set at *p* < 0.05.

## Abbreviations

FMOC: 9-Fluorenyl-methyl-chloroformate
BMI: Body mass index
BW: Body weight
BCAA: Branched chain amino acids
CRP: C-reactive protein
EHA: Elective hip arthroplasty
ESR: Erythrosedimentation rate
EAAs: Essential amino acids
HHS: Harris hip score
IGF-1: Insulin growth factor-1
OPA: Orthopthalaldehyde.
